# Evaluating models and assessment techniques for understanding oral biofilm complexity

**DOI:** 10.1002/mbo3.1377

**Published:** 2023-08-29

**Authors:** Srinivas Sulugodu Ramachandra, Patricia Wright, Pingping Han, Abdalla Abdal‐hay, Ryan S. B. Lee, Saso Ivanovski

**Affiliations:** ^1^ Centre for Orofacial Regeneration, Rehabilitation and Reconstruction (COR3), School of Dentistry, Faculty of Health and Behavioural Sciences The University of Queensland Brisbane Australia; ^2^ Preventive Dental Sciences, College of Dentistry Gulf Medical University Ajman United Arab Emirates; ^3^ School of Dentistry, Faculty of Health and Behavioural Sciences The University of Queensland Brisbane Australia; ^4^ Department of Engineering Materials and Mechanical Design, Faculty of Engineering South Valley University Qena Egypt; ^5^ Faculty of Industry and Energy Technology, Mechatronics Technology Program New Cairo Technological University, New Cairo‐Fifth Settlement Cairo Egypt

**Keywords:** assessment, biofilm, dynamic, polymicrobial, three‐dimensional model

## Abstract

Oral biofilms are three‐dimensional (3D) complex entities initiating dental diseases and have been evaluated extensively in the scientific literature using several biofilm models and assessment techniques. The list of biofilm models and assessment techniques may overwhelm a novice biofilm researcher. This narrative review aims to summarize the existing literature on biofilm models and assessment techniques, providing additional information on selecting an appropriate model and corresponding assessment techniques, which may be useful as a guide to the beginner biofilm investigator and as a refresher to experienced researchers. The review addresses previously established 2D models, outlining their advantages and limitations based on the growth environment, availability of nutrients, and the number of bacterial species, while also exploring novel 3D biofilm models. The growth of biofilms on clinically relevant 3D models, particularly melt electrowritten fibrous scaffolds, is discussed with a specific focus that has not been previously reported. Relevant studies on validated oral microcosm models that have recently gaining prominence are summarized. The review analyses the advantages and limitations of biofilm assessment methods, including colony forming unit culture, crystal violet, 2,3‐bis‐(2‐methoxy‐4‐nitro‐5‐sulfophenyl)‐2H‐tetrazolium‐5‐carboxanilide inner salt assays, confocal microscopy, fluorescence in situ hybridization, scanning electron microscopy, quantitative polymerase chain reaction, and next‐generation sequencing. The use of more complex models with advanced assessment methodologies, subject to the availability of equipment/facilities, may help in developing clinically relevant biofilms and answering appropriate research questions.

## Graphical abstract

This review focuses on diverse biofilm models and assessment strategies, highlighting the transition in the field from two‐dimensional models to the recently explored novel three‐dimensional models that aim to mimic biofilm development in the microenvironment of biological structures. The review further explores culture‐dependent biofilm assessment strategies such as colony‐forming unit assay, crystal violet assay, 2,3‐bis‐(2‐methoxy‐4‐nitro‐5‐sulfophenyl)‐2H‐tetrazolium‐5‐carboxanilide inner salt (XTT) assay, and imaging techniques, as well as the culture‐independent approach of next‐generation sequencing.

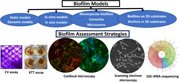

## 1 INTRODUCTION

Periodontitis and caries are common biofilm‐mediated dental diseases, which if untreated, may lead to loss of teeth (Chen et al., 2021; Wen et al., 2022). Even though the prevalence of caries is decreasing in the developed world, it remains high in developing and underdeveloped countries, making caries a global health challenge (Wen et al., 2022). Increased life expectancy, modern lifestyle with risk factors (smoking and stress), and increased retention of teeth have resulted in an increased global burden of periodontitis and it is expected to remain high (Tonetti et al., 2017). Although periodontitis is currently explained by the host–bacterial interaction model (Page & Kornman, 1997), dysbiotic microbiota existing in the oral cavity as biofilms initiate the disease (Ramachandra, Dopico, et al., 2017; Sanz et al., 2017). Oral bacteria are not planktonic, instead, they exist in the oral cavity as biofilms having additional virulence properties, including quorum sensing (Wright & Ramachandra, 2022), which protect them from the action of antimicrobials (Fernandez y Mostajo et al., 2011; Mombelli et al., 2011).

Of note, oral biofilms are distinctly different from biofilms observed elsewhere in the human body due to their unique location, dynamic nature, formation, and composition (Mosaddad et al., 2019). Oral biofilm which involves plaque formation on teeth/hard structures in the oral cavity is continuously forming, maturing, and is also disrupted by the oral hygiene practices of the individual. Biofilms observed in other parts of the body are mostly monospecies, for example, orthopedic and periprosthetic infections mostly contain *Staphylococcus epidermidis* and *Staphylococcus aureus (*Zimmerli & Sendi, 2017 *)*. On the contrary, biofilms formed in the oral cavity in different niches are known to be diverse with more than 700 bacterial species, as well as fungi, algae, and viruses (Wade, 2021). Some of the pathogenic bacteria seen in the oral cavity include *Streptococcus mutans*, *Porphyromonas gingivalis, Treponema denticola*, *Tannerella forsythia*, and *Aggregatibacter actinomycetemcomitans* (Socransky et al., 1998)*. P. gingivalis* is considered a keystone pathogen capable of driving the entire microbiome toward dysbiosis, resulting in periodontitis (Hajishengallis et al., 2012). Similarly, *S. mutans* is considered to be the key pathogen involved in the causation of smooth surface caries (Lemos et al., 2019). *Scardovia wiggsiae* is a novel pathogen isolated from the oral cavity implicated in the pathogenesis of early childhood caries (Kressirer et al., 2017). *S*everal other pathobionts also exist which are commensal under normal circumstances. However, under unfavorable circumstances like uncontrolled diabetes mellitus or smoking, the same bacteria can be pathogenic resulting in damage to the periodontium (Costalonga & Herzberg, 2014).

These biofilms are studied using several established in vitro and in vivo models and assessment methods (McBain, 2009). The list of these models and assessment methods is exhaustive and may overwhelm a novice biofilm researcher. Selecting an appropriate model and corresponding assessment technique are of paramount importance while designing research studies so that correct answers are derived for the framed research question (McBain, 2009). This review aims to summarize the existing literature on oral biofilm models and assessment methods. The review may serve as a useful guide for a beginner biofilm investigator and as a refresher for the established researcher. It assists in selecting appropriate models and assessment methodologies, thus designing scientifically sound biofilm studies.

## 2 BIOFILMS AND EXTRACELLULAR POLYMERIC SUBSTANCES (EPS)

### 2.1 Biofilms

**Figure 1:**
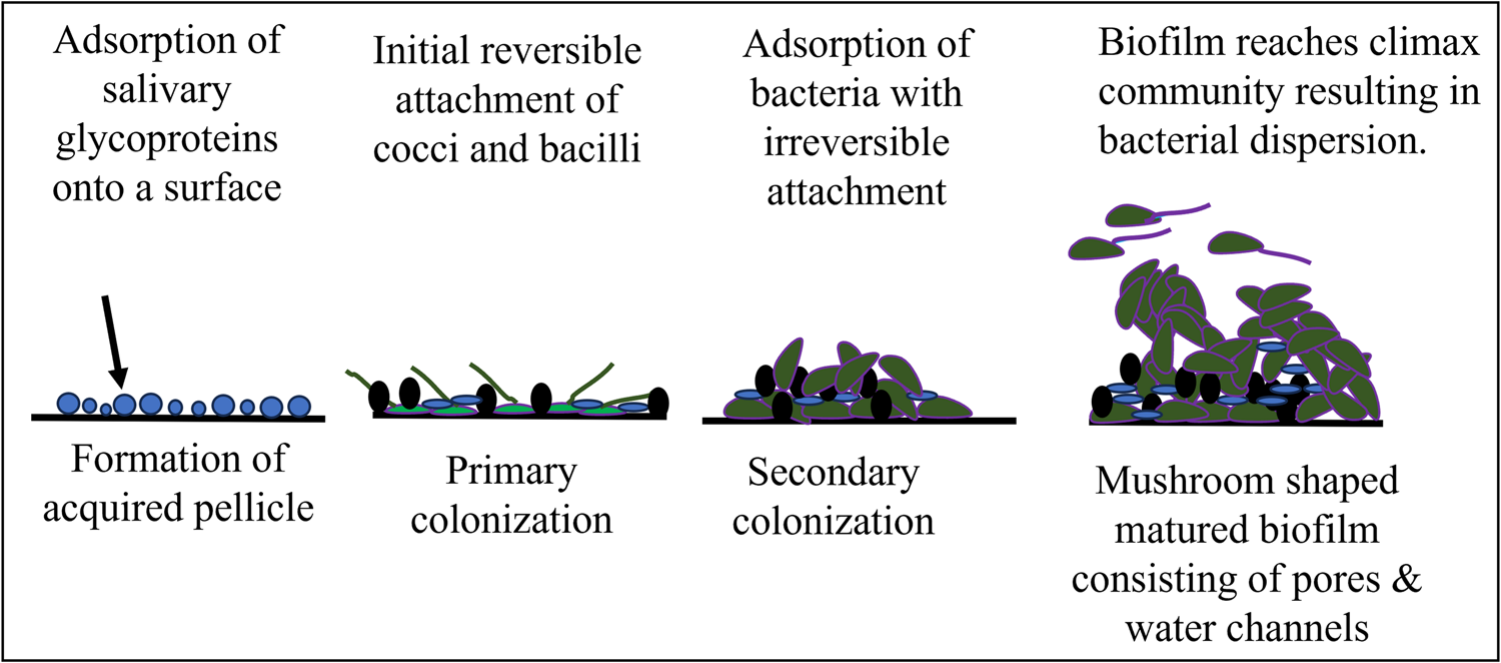
Schematic of a typical biofilm in different stages: Biofilm formation occurs in several stages. Within the oral cavity, in the initial stage, salivary glycoproteins adsorb onto the tooth surface, resulting in the formation of an acquired pellicle. Subsequently, aerobic bacteria including cocci and bacilli adhere to the tooth surface and the attachment is reversible. Later facultative anaerobic bacteria attach to each other and the surface of the existing biofilm matrix. Finally, a mature polymicrobial biofilm forms typical mushroom‐shaped towers with pores and water channels.

Biofilms are oriented aggregates of bacteria and/or yeast attached to a substrate and are entangled in a self‐produced matrix of extracellular polymer substances (EPS) (Hall‐Stoodley et al., 2004). Biofilms cause many infections in the human body including upper respiratory tract infections, urinary tract infections, otitis media, bacterial vaginitis, catheter‐induced infections, medical device/implant‐associated infections, periodontitis (Ramachandra, Gupta, et al., 2017), and dental caries (Costerton et al., 1999). Biofilm infections in immuno‐compromised individuals can lead to aggravation of disease and poor systemic health (Das et al., 2011). Biofilm formation occurs over several stages including the formation of an acquired pellicle, primary colonization of bacteria, secondary colonization of bacteria, and biofilm maturation. The different stages of biofilm development have been schematically depicted in Figure 1. Currently, a large number of medical/dental devices are implanted in humans (Kocak Oztug, 2021; Majid et al., 2022; Ramachandra et al., 2009). Many such devices end up with biofilm‐related infections, acting as a source for septicemia, necessitating their removal, and/or involving additional surgery or requiring the administration of antimicrobials (Arciola et al., 2018). Antimicrobials have limited penetration into biofilms due to the presence of EPS, rendering antimicrobials ineffective or partially effective (Singh et al., 2017). Inadequate concentrations of antimicrobials reach the inner layers of the biofilm, where bacteria may develop antimicrobial resistance (AMR) (Singh et al., 2017). Since many infections afflicting humans are biofilm‐related, and due to the possibility of the development of AMR, treating biofilm‐associated infections is a pressing challenge (Singh et al., 2017).

### 2.2 EPS

The two most important parts of a biofilm are the bacteria and the secreted matrix of EPS. In a mature biofilm, only 15%–20% of the volume is composed of bacteria, whereas the remaining 75%–80% is composed of EPS, which provides support for bacterial adherence, biofilm formation, and maintenance of biofilm integrity (Flemming, 2016). EPS contains extracellular polysaccharides, carbohydrate‐binding proteins, extracellular DNA, pili and flagella proteins, and other adhesive fibers secreted by the bacteria themselves (Flemming, 2016). EPS termed the “house of biofilms” offers protection to the bacteria from the host immune system and antimicrobials (Flemming et al., 2007). Recently, the focus of biofilm research has involved exploring the components of the EPS (Costa et al., 2020). Removal of the EPS and dispersal of the bacteria into a planktonic state may render the bacteria susceptible to antimicrobials and host immune cells (Costa et al., 2020). Table 1 lists the differences between bacteria in a planktonic state and a biofilm environment (Costerton et al., 1999). Thus targeting EPS in biofilms may provide solutions to biofilm‐related infections (Costa et al., 2020).

**Table 1:** Differences between planktonic bacteria and bacteria in a biofilm environment (Costerton et al., 1999; Schaudinn et al., 2009).

No	Bacteria in biofilms	Planktonic bacteria
1	Can cause chronic bacterial infections. For example: upper respiratory tract infections, urinary tract infections, otitis media, infection of medical and dental devices implanted in the body, periodontitis, chronic osteomyelitis, and dental caries.	Can cause acute bacterial infections. For example: Typhoid caused due to *Salmonella typhi*. *Escherichia coli* causes urinary tract infections. *Pseudomonas aeruginosa* causes lung infections in cystic fibrosis.
2	Attached to a surface or attached to each other.	Free‐floating bacteria.
3	Bacteria can increase their potency in response to environmental stimuli or adverse conditions by forming biofilms.	May be potent and capable of causing disease in a planktonic state.
4	Bacteria in biofilms secrete extracellular polysaccharides, proteins, lipids, and extracellular deoxyribonucleic acid that are collectively known as extracellular polymeric substances.	Do not secrete extracellular polymeric substances.
5	Biofilm bacteria are tolerant to the action of antimicrobials and host defense cells.	Are sensitive to the action of antimicrobials and host defense cells.
6	May develop antimicrobial resistance by various mechanisms.	Planktonic bacteria are sensitive to antimicrobials.
7	Following the maturation of the biofilm, bacterial aggregates may detach from the biofilm and form new biofilms.	Are usually localized, though their toxins may spill over to the bloodstream causing septicemia.
8	Bacteria in biofilms display regulation of gene expression by quorum sensing.	Planktonic bacteria do not display quorum sensing.

### 2.3 The role of oral biofilms in health and disease

Biofilms can be a friend and/or foe to the human body (Mangwani et al., 2016; Singh et al., 2006). On one hand, biofilms have beneficial properties in certain circumstances (Gutt et al., 2018). For example, commensal bacteria cause no damage to the oral tissues and are involved in the maintenance of oral health (Gutt et al., 2018). Some of these commensal bacteria are also primary colonizers, blocking the attachment of other pathogenic bacteria or pathobionts onto the tooth surface (Gutt et al., 2018), and are also involved in modulating the host response (Devine et al., 2015). On the other hand, biofilms also have destructive roles, helping the bacteria in evading host defense/action of antimicrobials and also playing a major role in the development of AMR (Singh et al., 2017).

### 2.4 Classification of biofilm models

**Figure 2:**
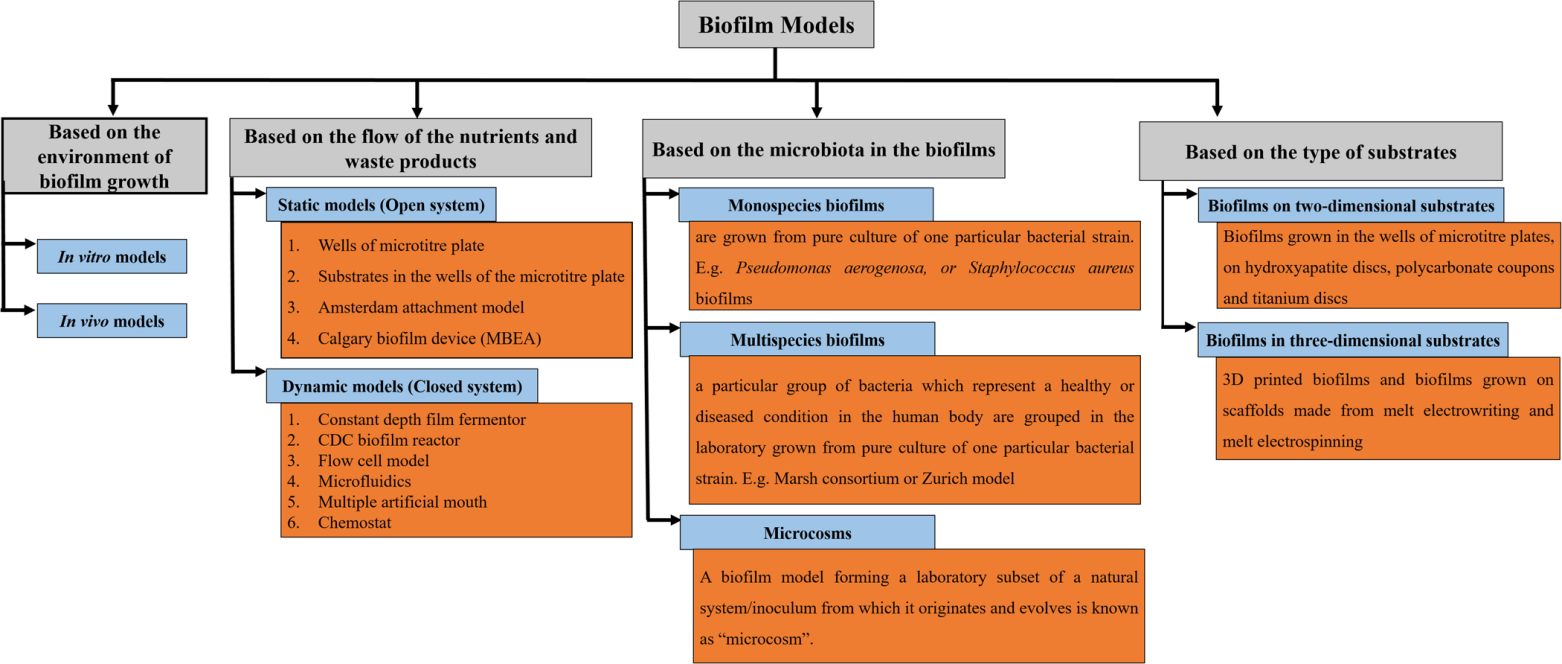
Biofilm models can be classified as “in vitro” and “in vivo” models (based on the environment of biofilm growth), static and dynamic models (based on the flow of nutrients and waste products), monospecies biofilms, multispecies biofilms, and microcosm models (based on the microbiota in the biofilms) and into biofilms in two‐dimensional and three‐dimensional models (based on the type of substrates).

Formation and growth of biofilms as well as the effect of antimicrobials on biofilms have been studied using several biofilm models (McBain, 2009). Thorough knowledge of biofilm models would help the researcher to select a suitable model based on the research design. These models are classified in several ways. Figure 2 summarizes the classification of biofilm models based on several variables as outlined below.

#### 2.4.1 Biofilm models can be classified as in vitro or in vivo models based on the environment of biofilm growth (Azeredo et al., 2017; Gabrilska & Rumbaugh, 2015)

(1) **In vitro models:** These laboratory models are designed to simulate clinical conditions and create artificial environments for the growth and study of biofilms (McBain, 2009). Even though many fungal and bacterial species are still not cultivable in an in vitro model, these models help study biofilms and the effect of oral healthcare products/antimicrobials on biofilms (Song et al., 2017). These models have several advantages including being easier to establish and inexpensive compared to in vivo models, providing high‐throughput data, and allowing control of several parameters like flow, amount of nutrients, and time points (Brown et al., 2019). Limitations include biofilms developed using these models do not mimic natural biofilms.
(2) **In vivo models:** Biofilms grown within appliances (appliances worn in the oral cavity by a volunteer/patient) in human beings or animal models are known as in vivo models (Costa et al., 2020). Advantages include that biofilms from these models mimic natural biofilms more closely, and are clinically relevant (Costa et al., 2020). Disadvantages include being expensive to establish and that projects need to satisfy a broad range of ethical requirements. Formation and development of biofilms are also dependent on volunteer/patient compliance.

#### 2.4.2 Biofilm models can be static (closed system), or dynamic (open system) models based on nutritional availability. Table 2 lists the differences between static and dynamic models (Azeredo et al., 2017; Brown et al., 2019; Gabrilska & Rumbaugh, 2015)

**Table 2:** Differences between static and dynamic models (Azeredo et al., 2017; Brown et al., 2019; Gabrilska & Rumbaugh, 2015).

No	Static models	Dynamic models
1	Biofilms are grown in an environment where the amount of nutrition is limited. Though there is an option for media replacement (nutrition) at timed intervals, nutrition is limited. These are also known as closed systems.	Biofilms are grown in an environment where nutrients are supplied/replenished constantly. These are also known as open systems.
2	Bacterial toxins or by‐products are retained in the same environment. This may enhance the growth of certain bacterial species and also suppress the growth of several others, thus altering the composition of the biofilm.	Bacterial toxins or by‐products are removed from the model constantly. The flow rate of fresh nutrient media into the model and the exit of waste media out of the model can be controlled.
3	Static models do not incorporate the shear forces that routinely occur in a natural biofilm environment. Even if movement is created, by, placing the static biofilm models on a shaker, the recreation of actual shear stress may not be possible.	Shear forces are created within the model, which results in the development of clinically relevant biofilm models.
4	Static systems are relatively inexpensive and not labor intensive.	Dynamic systems are expensive and labor‐intensive.
5	Example: Wells of a microtitre plate, substrates (hydroxyapatite discs, coverslips, titanium discs) placed in the wells of microtitre plates, or a Calgary biofilm device.	Examples: Flow cell biofilm system, center for disease control biofilm reactor; hollow fiber system; drip‐flow system.

(1) **Static models**: These models have limited nutrients within an enclosed environment, resulting in the rapid growth of biofilms without the removal of waste products (Brown et al., 2019). Shear forces exerted on the biofilms in static models are low or nonexistent, resulting in tower or mushroom‐shaped biofilms. Advantages include low cost, ease of establishment, and the ability to provide high throughput data (Brown et al., 2019). Minimal resemblance to natural biofilms and sedimentation of nutrients and waste products resulting in these products being incorporated within biofilms are some of the disadvantages (McBain, 2009).
(2) **Dynamic models**: have nutrients continuously fed into the model and permit waste product removal. Biofilms in these models are more aligned to clinical situations producing flatter and elongated biofilms compared to static models (Huang et al., 2023). These models allow the incorporation of shear forces during the experiment mimicking natural biofilms (Fernández et al., 2017; Sánchez et al., 2021). Some of the models are also equipped for real‐time imaging (Karygianni et al., 2014). Dynamic models are labor‐intensive, requiring large quantities of media, and are expensive to set up. Additionally, the systems provide only low data throughput (Sánchez et al., 2021).

#### 2.4.3 Biofilm models can be mono‐species or multi‐species or consortia, or microcosm models based on the number of bacterial species in the biofilm (Sánchez et al., 2021)

(1) **Mono‐species biofilms:** These biofilms contain only one bacterial species, also known as pure cultures. Mono‐species biofilms can be grown from strains of particular bacterial species procured from the American Type Culture Collection (ATCC), the National Collection of Industrial Food and Marine Bacteria (NCIMB), the German Collection of Microorganisms and Cell Cultures GmbH and other biotechnology companies (Collection, 2020; German Collection of Microorganisms and Cell Cultures GmbH, 2023; NCIMB, 2023). The study of mono‐species biofilms allows detailed evaluation of several characteristics as different variables can be controlled (McBain, 2009). However, the clinical relevance of these biofilms is limited as the majority of naturally occurring biofilms are polymicrobial (Brown et al., 2019).
(2) **Multispecies biofilms**: Biofilms containing two or more bacterial species are known as multispecies biofilms (Vila et al., 2021). Multispecies biofilms can also be created by growing fungi such as *Candida albicans* together with bacterial species, for example, *S. aureus* (Vila et al., 2021). However, in some of the published literature, these biofilms have been erroneously referred to as polymicrobial biofilms. This particularly applies in the context of oral biofilms which contain > 700 bacterial species (Wade, 2021). Biofilms grown from two or more bacterial species should be correctly referred to as multispecies biofilms rather than polymicrobial biofilms. A particular combination of bacterial species or a relationship between bacteria and fungi can be evaluated to explore plausible pathogenesis (Vila et al., 2021).
(3) **Consortia:** If a particular number of bacteria representing important physiological and ecological groups are grown together, such biofilms are known as consortia (McBain, 2009). For example, the Marsh consortium is a defined consortium that has been successfully employed in numerous studies, wherein approximately 10 oral bacteria that represent important physiological and ecological groups in the mouth are cultured (McKee et al., 1985). Sanchez et al. validated a biofilm model consisting of six standard bacterial strains mimicking the colonization and maturation of biofilms in the subgingival environment (Sánchez et al., 2011). The six bacterial strains incorporated were *Streptococcus oralis* and *Actinomyces naeslundii* (initial colonizers), *Veillonella parvula* (early colonizer), *Fusobacterium nucleatum* (secondary colonizer), and *P. gingivalis* and *A. actinomycetemcomitans* (late colonizers) (Sánchez et al., 2011). The Zurich model is another example of a consortium, where biofilms of *A. naeslundii, Veillonella dispar, F. nucleatum, Streptococcus sobrinus*, and *S. oralis* are grown on hydroxyapatite discs coated with salivary pellicle (Guggenheim et al., 2001). The plausible pathogenesis of a disease caused by a particular group of bacteria can be evaluated by studying a consortium of bacteria. However, reduced emphasis has been laid on studying consortia with the acceptance of the polymicrobial synergy and dysbiosis model (Hajishengallis & Lamont, 2012).
(4) **Microcosms:** Upon biofilm culture using natural body fluids (saliva, dental plaque, tears, and blood) as the inoculum, a minor portion (subset) of the microorganisms representing the original inoculum gets recreated in the formed biofilm. These are known as microcosms (McBain, 2009). Oral microcosms are a small subset of a dental plaque or salivary sample that is created in the laboratory for research purposes and which would contain bacteria, viruses, and fungi (Wong & Sissons, 2007). Rudney et al. validated microcosm biofilms grown on hydroxyapatite and composite discs from saliva or plaque by sequencing the genomic content in the biofilms (Rudney et al., 2012). The biofilms contained around 60% of the species in the original inoculum. Importantly, the addition of sucrose to the microcosms, induced changes conducive to the development of caries (Rudney et al., 2012). Sousa et al. developed an in vitro microcosm model in a constant depth film fermenter over 30 days, where the longitudinal shift of the bacteria from peri‐implant health to peri‐implant mucositis and peri‐implantitis was reported by 16S rRNA gene sequencing (Sousa et al., 2022). *Proteobacteria, Bacteroidetes, Firmicutes, Fusobacteria*, and *Actinobacteria* were the predominant phyla associated with peri‐implantitis (Sousa et al., 2022).

A significant similarity was observed in the microbial diversity of microcosms grown from saliva, subgingival plaque, tongue, and tonsils, indicating that saliva, tonsil swabs, and tongue scrapings can be alternate sources to grow microcosms in case it is cumbersome to collect subgingival plaque samples (Cieplik et al., 2019). The advantages of studying microcosms include the study of interspecies interactions, the possible synergistic and inhibitory effects between two different species within a microcosm, and the analysis of the effect of environmental changes on the biofilm (Diaz & Valm, 2020). A reduction in pH may enhance the caries risk, whereas an increase in pH in the subgingival environment may predispose the mouth toward periodontitis. Alterations in the microbial diversity due to changes in the pH and temperature can be studied in oral microcosm models. For example, the inclusion of arginine in an oral microcosm model increased the plaque pH despite the addition of sucrose, a supporting factor for the use of arginine‐based dentifrices in the prevention of dental caries (Ledder et al., 2017).

Evaluation of microcosms is clinically relevant and aligns with the polymicrobial synergy and dysbiosis model (Hajishengallis & Lamont, 2012). Limitations of studying microcosms are the requirements of specific nutritional media and growth environment (Rudney et al., 2012). Evaluation of microcosm models should include sequencing which may not be available at all localities.

#### 2.4.4 Biofilms can be grown on two‐dimensional (2D) and three‐dimensional (3D) substrates (Ramachandra et al., 2023). Table 3 lists the differences between biofilms cultured using 2D and 3D substrates

**Table 3:** Differences between biofilms grown using two‐dimensional (2D) and three‐dimensional (3D) substrates/models (Keleştemur et al., 2020; Ning et al., 2019; Ramachandra et al., 2023; Schaffner, 2017).

No	Biofilms grown on 2D models	Biofilms grown on 3D models
1	Supports biofilm growth in a horizontal dimension only	Supports biofilm growth in both horizontal and vertical dimensions
2	Biofilms grown on wells of microtitre plates, hydroxyapatite discs, titanium discs, polycarbonate coupons	Biofilms can be 3D printed, or biofilms grown in scaffolds obtained by melt electrowriting or melt electrospinning.
3	Biofilms are thin with less biovolume.	Thick biofilms are formed in a short time and the biovolume is more.
4	Less resistant to antimicrobials compared to biofilms in 3D substrates	More resistant to antimicrobials compared to biofilms in 2D substrates
5	Clinical relevance is less	More clinically relevant
6	Represents surface‐attached biofilms showing a mat of bacteria.	May represent submerged biofilms with bacteria crisscrossing the fibers

(1) **Biofilms on 2D substrates**: Traditional 2D substrates support biofilm growth primarily in the horizontal dimension only (Ramachandra et al., 2023). These substrates/models support the formation of thin biofilms with less biovolume. The biofilms produced by most 2D models have a thickness of < 100 µm. Despite attempts to mimic clinically relevant biofilms, these surface‐attached biofilms have shown limited success in replicating the complex architecture and microbial diversity found in natural biofilm communities (Ramachandra et al., 2023). The currently existing in vitro models have control over nutrients and fluid flow (Ning et al., 2019; Schmieden et al., 2018). However, these models do not reproduce a 3D microenvironment for clinically relevant biofilm growth (Ramachandra et al., 2023).
(2) **Biofilms in 3D substrates/models**: Biofilms have been traditionally studied using 2D substrates, although naturally occurring biofilms are 3D structures (Ramachandra et al., 2023). Hence, the biofilms developed from these 2D substrates are not representative of naturally occurring biofilms and are not clinically relevant (Ramachandra et al., 2023). Additionally, 2D substrates support biofilm growth only in the horizontal direction, whereas naturally occurring biofilms grow both horizontally and vertically. Over the past decade, cell culture studies have been performed on 3D substrates (Heuer et al., 2022). Only recently attempts have been made to 3D print biofilms (Balasubramanian et al., 2019) and grow biofilms in 3D melt electrowritten scaffolds (Ramachandra et al., 2023). Melt electro writing (MEW) can create controlled and highly ordered 3D geometries resembling the microanatomy of biological structures (Ramachandra et al., 2023). Recently, researchers suggested fabricating novel compact biofilm models that are dense, and at the same time, closely and orderly packed together (Guzmán‐Soto et al., 2021). The report also suggested including features that better mimic the target microenvironment (Guzmán‐Soto et al., 2021). Since MEW can create high‐fidelity scaffolds that mimic structured microenvironments, the additive manufacturing technique can be utilized to fabricate novel biofilm models.

**Figure 3:**
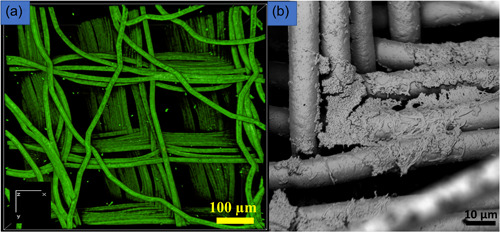
(a) Confocal image of a 3D melt electrowritten scaffold stained with SYTO 9 (calibration *x*: 2.47 µm, *y*: 2.47 µm, *z*: 15.00 mm). Biofilms were imaged using a confocal laser microscope (Nikon Eclipse Ti confocal Microscope) at 488 and 561 nm. These scaffolds can provide niches for bacteria to grow both in vertical and horizontal dimensions, additionally creating a 3D microenvironment. (b) Scanning electron microscope image of salivary biofilms grown in 3D melt electrowritten scaffolds. The bacteria are seen crisscrossing the fibers of the scaffolds. From data collected during research work towards the degree of Doctor of Philosophy, The School of Dentistry, The University of Queensland. Herston, Australia. https://doi.org/10.14264/b9fe031.

MEW 3D scaffolds support biofilm growth both in horizontal and vertical dimensions (Ramachandra et al., 2023). These biofilms may resemble submerged biofilms and are more clinically relevant compared to biofilms on 2D substrates (Ramachandra et al., 2023). Thicker biofilms with higher biovolume and substratum coverage can be produced more quickly compared to biofilms on 2D substrates (Ramachandra et al., 2023). Membranes/soft tissue grafts (Ramachandra et al., 2014) and 3D constructs/scaffolds are used for periodontal and bone regeneration (Ivanovski et al., 2014). The use of membranes for regenerative purposes is known as “guided tissue regeneration,” (Ma & Yan, 2023) whereas the use of scaffolds for regeneration has been termed “scaffold‐guided regeneration” (Bartnikowski et al., 2020; Laubach et al., 2022). However, these membranes/soft tissue grafts and scaffolds may be exposed to the oral cavity leading to possible biofilm contamination (Abdo et al., 2023; Rasperini et al., 2015). Thus, biofilms grown on these porous additively manufactured 3D scaffolds can be models to study biofilm‐contaminated 3D constructs or membranes/soft tissue grafts (Ramachandra et al., 2023). Additive manufacturing technologies offer opportunities for fabricating 3D substrates for biofilm modeling. Biofilms that are 3D printed (Schaffner, 2017), or cultured in scaffolds obtained by MEW (Ramachandra et al., 2023), or melt electrospinning can recreate a 3D microenvironment (Figure 3a,b) (Keleştemur et al., 2020). Vertically aligned calcium phosphate nanoplates coated on 3D melt electrowritten scaffolds may inhibit biofilm formation, thus providing possibilities for creating scaffolds with antibacterial properties (Abdal‐hay et al., 2023). Recently, a study reported modifying a commercial 3D printer with a bio‐ink containing engineered bacteria (Ning et al., 2019). *E. coli* bacteria were suspended in a solution of alginate mixed with brain heart infusion broth. Other 3D bacterial biofilm constructs utilized bacteria including methicillin‐resistant *S. aureus* (MRSA), methicillin‐sensitive *S. aureus* (MSSA), and *Pseudomonas aeruginosa*. The thickness of the biofilms formed varied from 0.25 to 4 mm and showed greater AMR compared to biofilms grown on 2D substrates (Ning et al., 2019). 3D‐printed biofilms were also found to have increased AMR compared to biofilms on 2D substrates (Spiesz EM et al., 2019). However, producing such 3D‐printed biofilms needs a dedicated 3D printer for bacterial work (Ning et al., 2019).

Although various models are used to generate biofilms, the bacterial diversity of these biofilms, especially for in vitro oral multispecies biofilms, is dependent on the age of the biofilms, the media composition, frequency of media changes, incubation conditions (aerobic vs. anaerobic), source of the inoculum and shear forces (Fernández et al., 2017).

### 2.5 Currently available in vitro biofilm models

Most of the current knowledge regarding biofilms has been derived from studying biofilms grown using in vitro biofilm models (Hall‐Stoodley et al., 2004). The following paragraphs describe the features of commonly used biofilm models.

#### 2.5.1 Static biofilm models

(1) **Wells of microtitre plates**: made of polystyrene are inexpensive and have high throughput. However, bacterial byproducts may sediment to the bottom of the plates. This is considered a disadvantage (Azeredo et al., 2017). Bacterial byproducts of one species can affect the growth and development of other bacterial species, especially in a polymicrobial biofilm. In static models, the bacterial byproducts can accumulate as sediments, which can either inhibit or accelerate the growth and development of other bacteria (Azeredo et al., 2017).
(2) **Substrates in the wells of the microtitre plates**: Biofilms form on all surfaces and hence a large number of materials have been used as substrates to grow biofilms in the laboratory (McBain, 2009). These substrates include hydroxyapatite discs, cover glasses, coupons of titanium and alloys of titanium (Mendhi et al., 2021), polycarbonate coupons, polymethylmethacrylate, stainless steel, and other substrates that can be placed in the wells of the microtitre plates to study the growth of biofilms. However, these models still suffer from sediments settling at the bottom of the well.
(3) **Calgary Biofilm Device**: Ceri et al. at the University of Calgary developed a model, with a plastic lid having 96 pegs and a corresponding base (Ceri et al., 1999). The plastic lid with the pegs fits into the wells of a 96‐well microtitre plate. Biofilms grown on the pegs constitute attached biofilms without sediments. The pegs can be separated from the base of the lid to evaluate the effect of antimicrobials on the biofilms (Ceri et al., 1999). This model was named the “Calgary Biofilm Device” (Ceri et al., 1999), and it is currently marketed as the minimal biofilm eradication concentration (MBEC) assay (Ceri et al., 1999).
(4) **Amsterdam Active Attachment model (**Exterkate et al., 2010 **)**: In this model, biofilms are grown on substrates held by pegs, like the Calgary biofilm device. However, biofilms can be grown on different substrates fixed to the pegs of the device (Exterkate et al., 2010).

#### 2.5.2 Dynamic biofilm models

**Figure 4:**
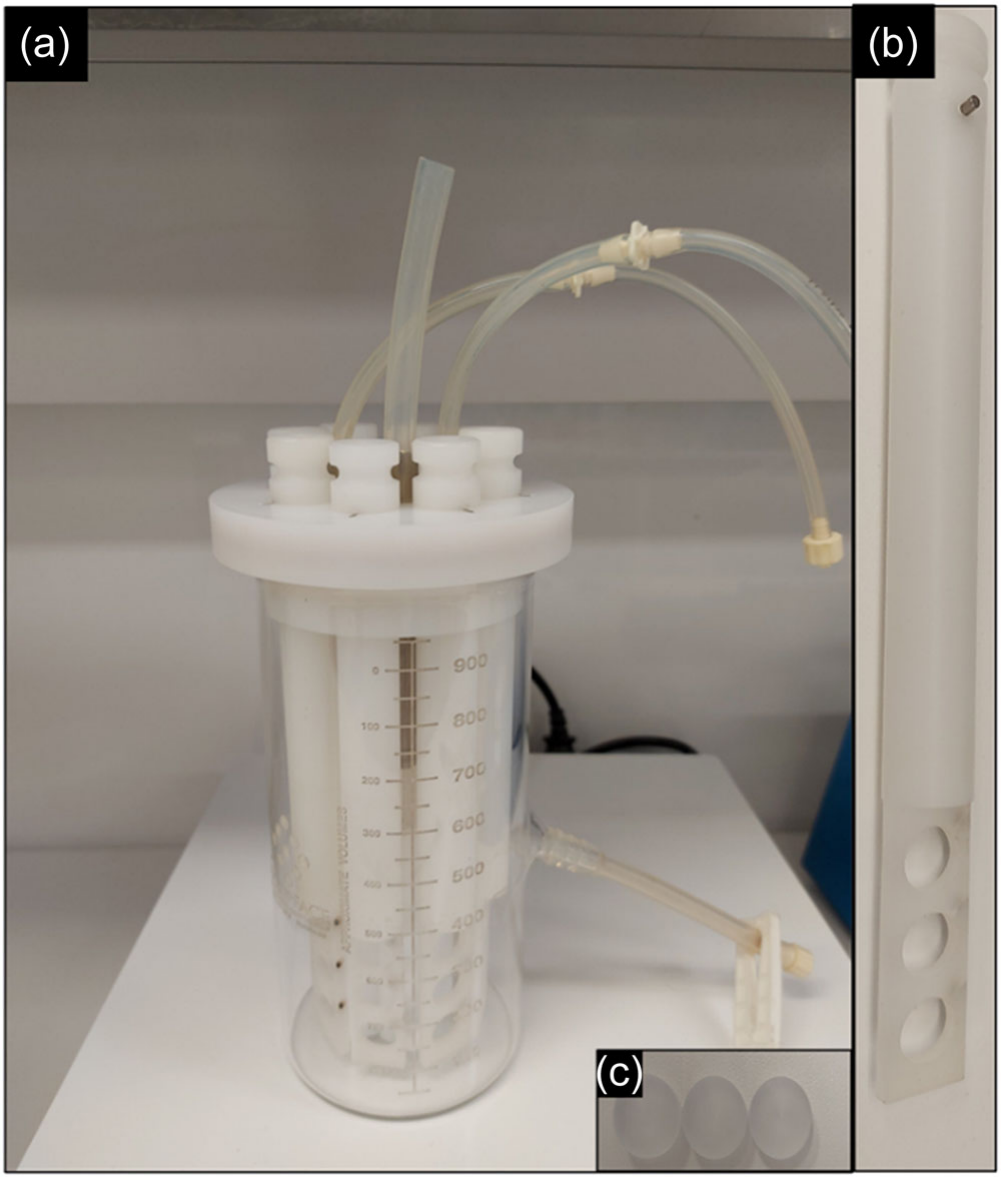
CDC biofilm reactor has eight polypropylene coupon holders (a), with each rod having three slots for placement of coupons (b). Biofilms grow on coupons that fit into slots within these rods (c). CDC, Centre for Disease Control.

**Figure 5:**
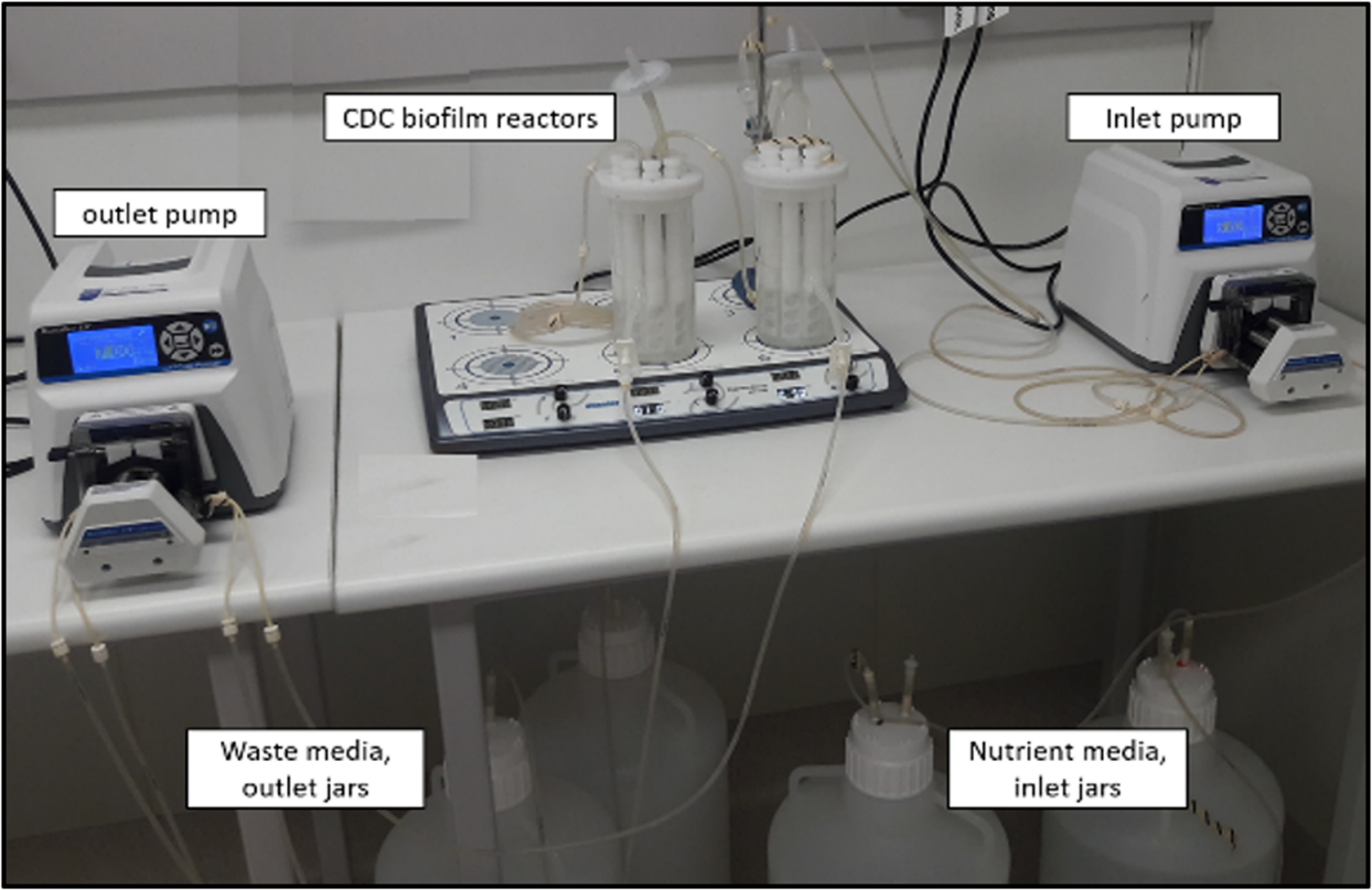
Image of a setup for a CDC biofilm reactor infection model. Nutrient media is pumped through the inlet pump into the CDC biofilm reactor and an outlet pump removes waste media into the waste jars. CDC, Centre for Disease Control.

(1) **Constant depth film fermenter (CDFF)**: The model has a static scraper blade, against which a stainless‐steel disk (holding the sample) rotates. The scraper blade removes excess biofilm, thus reproducing and maintaining a constant depth of biofilm. The CDFF model is advantageous because it supports restrained growth and can produce oral biofilms of constant thickness (Pratten, 2007; Wilson, 1999). As the thickness of biofilms is predetermined, subsample and effluent analysis are limited to some extent. The thickness of biofilms grown in a CDFF device is 50–500 µm. Oral biofilms are around 200 µm thick (Wood et al., 2000), so by using a CDFF, biofilm thickness is maintained in that particular range (Roberts et al., 2015).
(2) **Flow‐cell systems**: The scientific literature considers flow‐cell systems as the gold standard among various biofilm models to observe the various stages of biofilm formation in a noninvasive manner (Crusz et al., 2012; Sternberg & Tolker‐Nielsen, 2006). In flow‐cell systems, bacteria are grown in small fluid‐filled channels, through which biofilms are monitored in real‐time. The growth of the biofilms is checked non‐invasively using direct microscopy. However, flow‐cell systems allow observation of only a few samples at one time and the equipment is expensive (Crusz et al., 2012). The time required to complete one experiment is longer than that for a microtitre plate.
(3) **Center for Disease Control (CDC) biofilm reactor**: is a dynamic biofilm model produced by the Center for Biofilm Engineering, at the Montana State University (Goeres et al., 2005). It consists of a 1‐L glass vessel with a side‐arm discharge port at around 350 ml. The vessel has a lid that can suspend eight polypropylene rods with slots for holding coupons (Figure 4). Each rod has three slots for the placement of coupons that are tightened using small screws (Rudney et al., 2012). A long list of coupons made from different materials is available. A baffle present in the vessel is rotated via a magnetic stirrer plate to generate moderate and high shear stress. Nutrient media is circulated at a defined rate through the reactor using a pump (Figure 5). Similarly, the waste media is removed from the reactor using another pump (Figure 5). Antimicrobials can be introduced into the reactor using one of the ports at a desired infusion rate mimicking various routes of drug administration (Williams et al., 2019). Evaluation of the effect of antimicrobials on biofilms is performed by retrieving the coupons at various time points from the reactor. Aerobic and anaerobic biofilm reactors are available for the growth of biofilms of different bacteria (Rudney et al., 2012; Song et al., 2017).
(4) **Microfluidics:** This model uses miniaturized channels with laminar (unidirectional) fluid flow for biofilm growth (Neethirajan, 2012). The model allows for the control of fluid flow rate and shear stresses, which are important factors that control the thickness, density, and surface coverage of biofilms (Nance et al., 2013). The system allows for real‐time imaging of the biofilms using confocal microscopy (Holman et al., 2009).
(5) **Multiple artificial mouth:** Artificial mouth model for developing oral microcosms have been in use since 1991 (Sissons et al., 1991). The latest advances are comprehensive biofilm models that incorporate computerized systems which control the various factors and variables affecting biofilm growth, pH, metabolism, and mineralization (Qamar et al., 2022).
(6) **Chemostat:** These models use chambers to grow biofilms in dynamic conditions with regular feeding of nutrient media and removal of waste products (Qamar et al., 2022). Several modifications of chemostats have been used to grow biofilms (Bradshaw et al., 1996).

## 3 BIOFILM ASSESSMENT METHODS

Qualitative and quantitative methods are used to assess biofilm growth (Azeredo et al., 2017). Some of the qualitative data can be subsequently analyzed quantitatively if a sufficient sample size allows for quantitative analysis (Wright et al., 2021). Each method has advantages and disadvantages. A research design with only one assessment method, may not be the best research design (Wright et al., 2021). A combination of qualitative and quantitative assessment methods would sufficiently address specific research questions (Azeredo et al., 2017). Table 4 lists an overview of the two broad types of biofilms and the corresponding assessment methods that should be used to appropriately answer the research question.

**Table 4:** The different types of biofilms, the models that could be used, and the corresponding assessment methods that can be employed to evaluate them.

Types of biofilms	Models that could be used	Assessment methods	
		Quantitative	Qualitative
− Mono‐species biofilms, − Consortia	Static or dynamic models	Crystal violet assay XTT assay Colony forming units Quantitative polymerase chain reaction with specific primers	Live‐dead imaging using confocal microscopy* Scanning electron microscopy*
− Polymicrobial biofilms − Microcosms	Preferably dynamic models	Crystal violet assay XTT assay Next‐generation sequencing and metagenomics	Live‐dead imaging using confocal microscopy Fluorescence In Situ Hybridization Confocal microscopy* Scanning electron microscopy*

Abbreviation: 2,3‐bis‐(2‐methoxy‐4‐nitro‐5‐sulfophenyl)‐2H‐tetrazolium‐5‐carboxanilide inner salt.

* Confocal images and Scanning electron microscopic images can be quantified using image processing software.

### 3.1 Colony‐forming units (CFUs)

Biofilms are dispersed by vortexing and sonication, then plated on an agar plate for incubation, and quantification (Magana et al., 2018; Welch et al., 2012). This is a commonly employed method of biofilm assessment and it is inexpensive. However, sonication and vortexing for biofilm dispersal will destroy the biofilm structure. Hence, it is not a good method to observe the structure of biofilms. Additionally, the process of sonication and vortexing may kill bacteria, restricting any further evaluation of the biofilm. This method allows the assessment of only cultivable bacteria, which may constitute only 5% of the original bacterial inoculum (Magana et al., 2018). Therefore, a significant portion of bacteria is not evaluated, a concept known as the “great colony count anomaly” (Tanaka et al., 2014; Váradi et al., 2017).

### 3.2 **Crystal violet (CV) assay (**Xu et al., 2016 **)**

Biofilms are stained using CV stain to quantitatively assess the total biomass of the biofilms. The biomass consists of live and dead bacteria in addition to the extracellular matrix (Magana et al., 2018). Though it is a commonly used assessment method, the assay does not discriminate between vital and dead bacteria. Additionally, the CV stain may bind unspecifically to negatively charged molecules, resulting in uneven extraction of the dye from the biofilms. Therefore, the assay lacks sensitivity and specificity (Magana et al., 2018).

### 3.3 **XTT assay (**Xu et al., 2016 **)**

2,3‐bis‐(2‐methoxy‐4‐nitro‐5‐sulfophenyl)‐2H‐tetrazolium‐5‐carboxanilide inner salt (XTT) assay quantitatively measures the metabolic activity of biofilms. XTT is a tetrazolium sodium salt that gets cleaved by dehydrogenase enzymes that are only present in metabolically active bacteria. This results in the formation of a highly colored formazan product, the optical density of which is measured spectrophotometrically (Koban et al., 2012). This inexpensive assay is highly sensitive for measuring cell viability and is suitable for antimicrobial testing (Xu et al., 2016). The assay does not disturb the biofilms structurally and the results of the assay are available within 3–4 h. However, different stains and species may metabolize XTT differentially, resulting in assessment inaccuracies in multispecies biofilms (Kuhn et al., 2003).

### 3.4 Confocal laser scanning microscopy (CLSM) and image quantification

**Figure 6:**
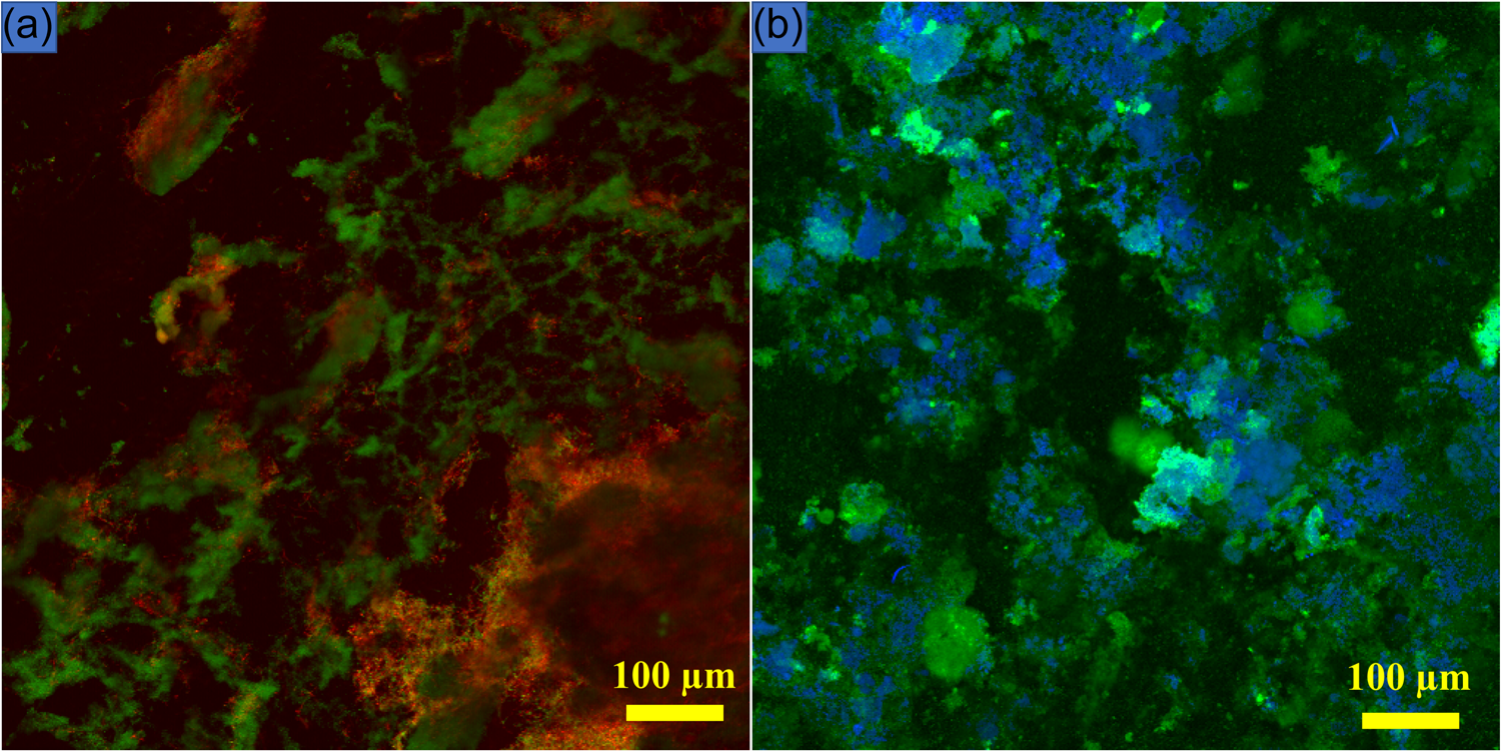
Various stains can be used to visualize biofilms using confocal microscopy and specific dyes/molecular probes. (a) A confocal microscopy image of a 5‐day‐old subgingival plaque microcosm treated with amoxicillin stained with SYTO 9 and propidium iodide (LIVE/DEAD^TM^ BacLight^TM^ Bacterial Viability Kit). Green fluorescence indicates live bacteria, whereas red fluorescence indicates dead bacteria. (b) A confocal image of a 10‐day‐old salivary microcosm biofilm grown on a hydroxyapatite disc in an anaerobic box at 37°C stained with SYTO 9 (bacteria stained green) and calcofluor‐white (extracellular matrix of the biofilms stained blue) (calibration *x*: 2.47 µm, *y*: 2.47 µm). Biofilms were imaged using a confocal laser microscope (Nikon Eclipse Ti confocal Microscope) at 488 and 561 nm. From data collected during research work towards the degree of Doctor of Philosophy, The School of Dentistry, The University of Queensland, Herston, Australia. https://doi.org/10.14264/b9fe031.

A major advancement in biofilm research was the use of CLSM in the early 1990s (Lawrence et al., 1991). One of the mandatory requirements for the initiation and formation of biofilms is the presence of moisture/water. A series of 2D images is recorded, which are later used for the reconstruction of 3D images. CLSM allows visualization of biofilms in their natural hydrated environment. The technique creates sectioned images at depths of micrometers, allowing detailed study and depth assessment (Karygianni et al., 2014). Biofilm thickness, an important parameter, can be measured. Evaluation of specific bacteria is possible by tagging with particular fluorescence proteins (Karygianni et al., 2014). A commonly employed method involves quantifying live and dead bacteria (Cheng et al., 2016), using various molecular probes like SYTO 9 and propidium iodide (Figure 6a) (Zhang et al., 2019). The use of SYTO 9 and propidium iodide for live‐dead staining has been criticized owing to the bleaching effect of SYTO 9, and possible background fluorescence impacting overall results (Netuschil et al., 2014; Shi et al., 2007; Stiefel et al., 2015). Despite the possible confusion over their usage, they continue to be utilized and reported frequently in the current biofilm literature (Mendhi et al., 2021).

Using stains like dextran conjugated Alexa Fluor (Philip et al., 2019) and calcofluor white (Grecka et al., 2020) (Figure 6b), the biofilm matrix can also be visualized (Zhang et al., 2019). Several image analysis software programs are available including COMSTAT (Heydorn et al., 2000), IMARIS, Image J (Schindelin et al., 2012), and BiofilmQ (Hartmann et al., 2021) that can quantify confocal images. Limitations include the high cost of equipment and the associated molecular probes. Additionally, high‐quality confocal imaging has a learning curve.

### 3.5 Fluorescence in situ hybridization (FISH)

This technique can visualize and quantify the specific location of the microbial species within polymicrobial biofilms or microcosms (Karygianni et al., 2014). Often this technique is combined with confocal microscopy to gain valuable insights into the spatial organization and the composition of microbial species within polymicrobial biofilms (Karygianni et al., 2014).

### 3.6 Scanning electron microscopy (SEM)

**Figure 7:**
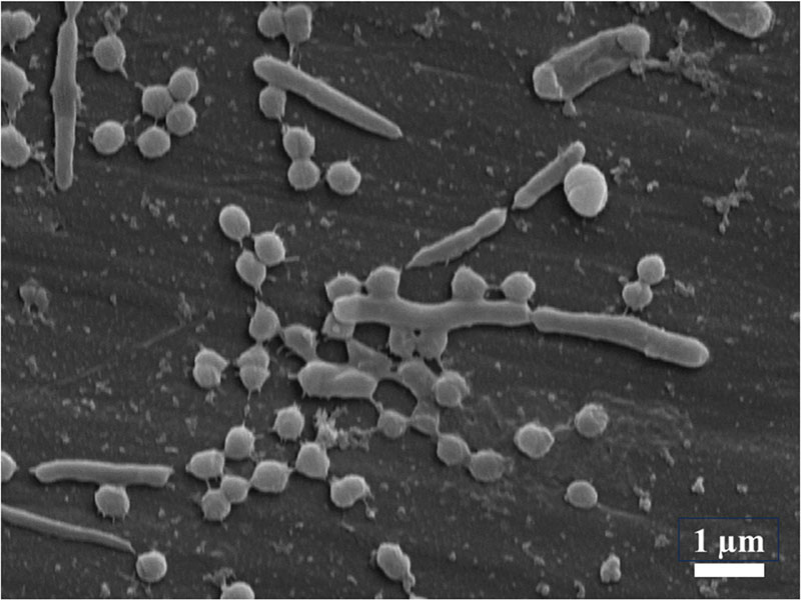
Scanning electron microscopic image of a 2‐day salivary microcosm biofilm on a two‐dimensional medical grade polycaprolactone film grown in a microtitre plate in an anaerobic box at 37°C. The inoculum used consisted of 85% brain heart infusion, 5% defibrinated sheep's blood, and 10% pooled saliva from six periodontally healthy volunteers. Cocci and rod‐shaped bacilli are present. From data collected during research work towards the degree of Doctor of Philosophy, The School of Dentistry, The University of Queensland, Herston, Australia. https://doi.org/10.14264/b9fe031.

SEM is used to visualize the surface biofilm topography (Figure 7). To visualize the samples under SEM, they are processed using fixatives such as glutaraldehyde and dried with graded concentrations of ethanol, which may damage the structure of the EPS and cause shrinkage of the cells (Azeredo et al., 2017). SEM allows the visualization of bacterial cells and EPS at micrometer and nanometer levels (Danilova et al., 2018; Xiang et al., 2022). SEM has several limitations including the sample processing being invasive, which may result in shrinkage and alteration of bacterial morphology (Costa et al., 2020) and the fixation processes that kill bacteria, making it impossible to distinguish between vital and nonvital bacteria. The high cost of the equipment and the necessity of trained personnel for equipment maintenance are additional disadvantages.

### 3.7 Molecular biological methods for biofilm assessment

Quantitative polymerase chain reaction (qPCR) (Suzuki et al., 2005) and next‐generation sequencing (NGS) are two methods that are culture‐independent and are utilized in assessing biofilms (Conrads et al., 2019; Tanaka et al., 2014).

#### 3.7.1 qPCR

In qPCR, bacterial primers complementary to nucleotide sequences present in the biofilm are used to identify known bacteria (Suzuki et al., 2005). Distinct bacterial primers are essential for each target. qPCR is a highly sensitive assay that provides quick and high‐throughput detection of DNA sequences (Swimberghe et al., 2019). qPCR is unable to distinguish between live and dead bacterial genomic DNA. Subsequently, the combination of propidium monoazide and qPCR was used to identify live bacteria (Brauge et al., 2019). qPCR has been criticized for being an assay with a narrow approach that identifies bacteria with specific primers (Brauge et al., 2019). It is estimated that > 700 bacterial species are present within the oral cavity (Wade, 2021). Identifying only 10–20 specific species may not reveal the true microbial diversity, especially if microcosms or polymicrobial biofilms are being studied. During the assay, primers may amplify species other than the target resulting in biased results.

#### 3.7.2 NGS

**Figure 8:**
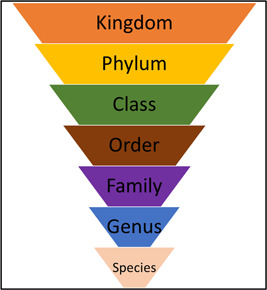
A schematic image depicting the hierarchy and the most used levels in bacterial taxonomy. Following next‐generation sequencing, bacteria are presented as kingdom, phylum, class, order, family, genus, and species.

**Figure 9:**
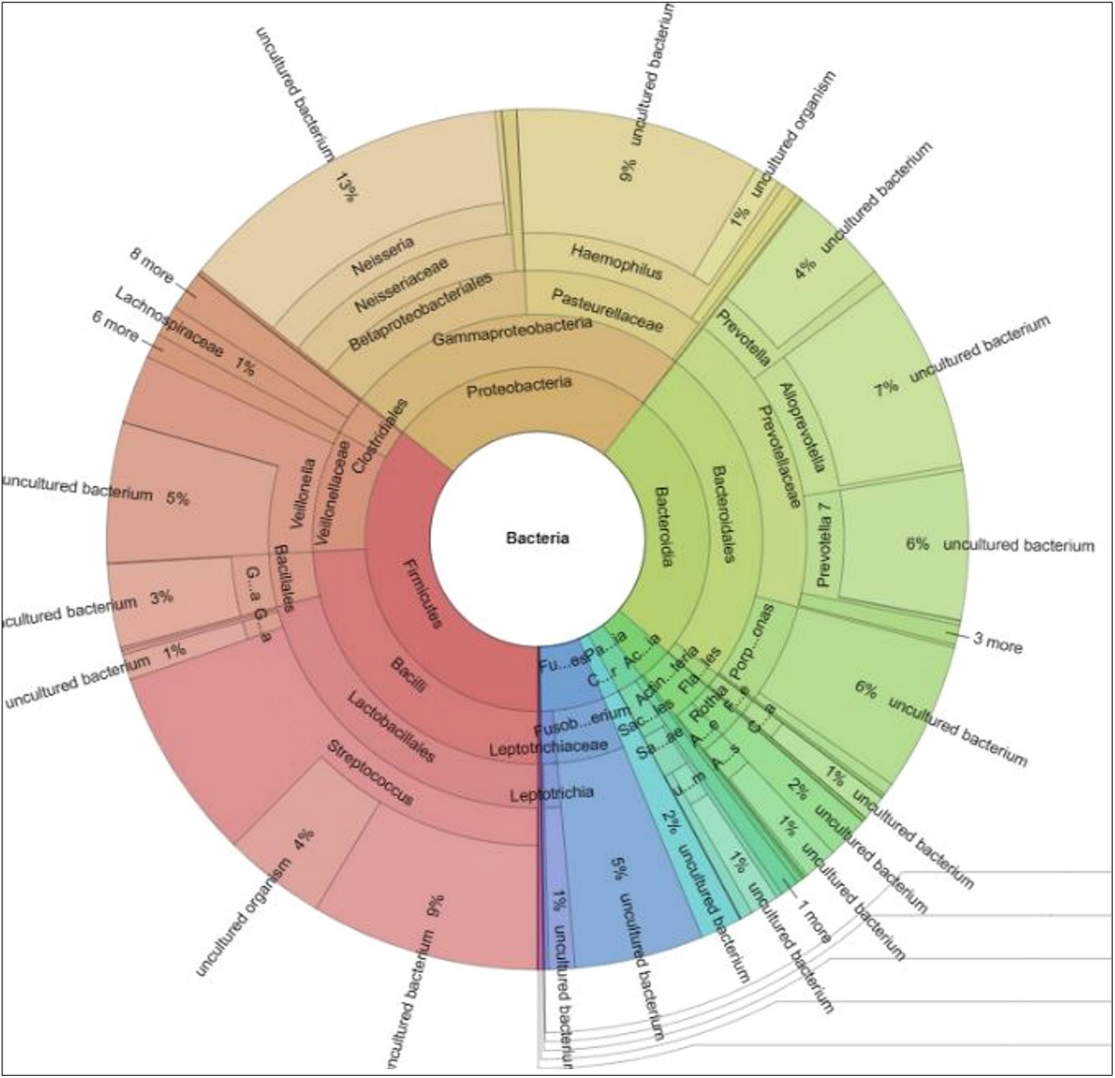
A representative Krona plot depicting the salivary microbiome following 16S rRNA gene sequencing. Unstimulated saliva was collected from six periodontally healthy individuals, and genomic DNA was isolated and subsequently 16S rRNA gene sequencing was carried out. Next‐generation sequencing is a culture‐independent method to qualitatively assess the microbiome. Sequencing provides information on cultivable and noncultivable bacterial species in a biofilm. From data collected during research work towards the degree of Doctor of Philosophy, The School of Dentistry, The University of Queensland, Herston, Australia. https://doi.org/10.14264/b9fe031.

Identification of uncultivable bacteria and new bacterial species is possible using the broader approach of NGS which is dependent upon the 16 S rRNA gene (Verma et al., 2018). Sequencing provides information on the microbiome using the levels of bacterial taxonomy (Figure 8) and most platforms provide an easily comprehensible Krona plot which provides detailed information about the microbiome in any inoculum (Figure 9). With the advent of 16S rRNA gene sequencing, the whole microbiome present in biofilms has been sequenced (Verma et al., 2018). NGS can identify bacteria that do not divide and instead exist in low metabolic states. These are known as viable but noncultivable bacteria (VBNC) and retain the capacity to be cultivable once resuscitated (Oliver, 2010; Pinto et al., 2015). Limitations of the approach include the cost involved in processing the samples and the necessary sequencing facilities may be unavailable in certain locations.

## 4 CONCLUSION AND FUTURE DIRECTIONS

In conclusion, this review provides an overview of the various biofilm models and assessment methods used in oral biofilm research. The review emphasizes the need for specific assessment methods to be employed based on the research design of the study. While quantitative methods are useful for understanding the bacterial load, qualitative methods such as confocal and scanning electron microscopy are important for visualizing biofilm architecture and distribution. New generation 3D models have been recently explored which create a 3D microenvironment and are more clinically relevant. The study of oral biofilms in 3D models may reveal more information about the structure within biofilms and possibly answer some of the questions surrounding biofilm‐related infections.

Novel whole genome sequencing technologies can provide in‐depth information about the microbial community within biofilms, enabling researchers to better understand the mechanisms of biofilm formation and the interactions between microorganisms. The continued development and application of these technologies will further our understanding of the composition of biofilms and their role in oral diseases, paving the way for more effective prevention and treatment strategies. Multidisciplinary research involving various fields may pave the way forward for oral biofilm research.

## AUTHOR CONTRIBUTIONS

**Srinivas Sulugodu Ramachandra**: Conceptualization (equal); data curation (equal); methodology (equal); visualization (lead); writing—original draft (lead); writing—review and editing (lead). **Patricia Wright**: Conceptualization (supporting); methodology (equal); writing—original draft (supporting); writing—review and editing (lead). **Pingping Han**: Conceptualization (supporting); data curation (supporting); methodology (supporting); supervision (lead); writing—original draft (supporting); writing—review and editing (supporting). **Abdalla Abdal‐hay**: Conceptualization (supporting); methodology (supporting); supervision (supporting); writing—original draft (supporting); writing—review and editing (supporting). **Ryan S. B. Lee**: Conceptualization (equal); methodology (supporting); supervision (supporting); writing—original draft (supporting); writing—review and editing (supporting). **Saso Ivanovski**: Conceptualization (equal); methodology (equal); resources (equal); supervision (lead); writing—original draft (supporting); writing—review and editing (supporting).

## CONFLICT OF INTEREST STATEMENT

None declared.

## ETHICS STATEMENT

None required.

## Data Availability

Not applicable.
